# Negr1-Derived Peptides Trigger ALK Degradation and Halt Neuroblastoma Progression In Vitro and In Vivo

**DOI:** 10.3390/pharmaceutics15092307

**Published:** 2023-09-12

**Authors:** Francesca Pischedda, Alessia Ghirelli, Vasvi Tripathi, Giovanni Piccoli

**Affiliations:** Department of Cellular, Computational and Integrative Biology—CIBIO, University of Trento, 38123 Trento, Italy

**Keywords:** Negr1, IgLON, neuroblastoma, peptide, therapy

## Abstract

Neuroblastoma is among the most common childhood cancers. Neuroblastoma in advanced stages is one of the most intractable pediatric cancers, notwithstanding the recent therapeutic advances. ALK mutations are among the leading cause of hereditary neuroblastoma and account for more than 14% of the somatically acquired alterations. ALK kinase activity is currently one of the main targets for pharmacological strategies. However, evidence from ALK fusion-positive lung cancer studies has shown that resistance to ALK inhibition arises during the therapy, causing a relapse within several years. IgLONs are membrane-bound proteins involved in cell-to-cell adhesion. The expression of the IgLON family results altered in different cancers. We found that the IgLON member Negr1 is downregulated in neuroblastoma. The ectopic overexpression of Negr1 impairs neuroblastoma growth in vitro and in vivo. Negr1 exists as a GPI-anchored membrane-bound protein and as a soluble protein released upon metalloprotease cleavage. We generated and characterized a panel of Negr1-derived peptides. The treatment with Negr1 protein and derived peptides induce ALK downregulation and halt neuroblastoma progression in vitro and in vivo.

## 1. Introduction

Neuroblastoma (ORPHA: 635) is the most common extra-cranial childhood cancer and represents about 10% of solid tumors in infants and children under the age of 15, with an annual incidence of about 1/70,000 in children in this class of age. Neuroblastoma can affect the sympathetic nervous system, such as the paraspinal ganglia or adrenal medulla, and generate a noticeable mass in the chest, neck, pelvis, or abdomen [1]. Clinically, neuroblastoma is divided into low- and high-risk, depending on the age at diagnosis, stage, tumor histology, MYCN status, and tumor cell ploidy [2]. Surgical intervention alone or in combination with minimal chemotherapy treatment can increase survival in low-risk cases. The 5-year survival rate in low-risk patients is 85–90%, but it drops to 50% in the high-risk ones [3,4]. Indeed, neuroblastoma in advanced stages is one of the most intractable pediatric cancers, notwithstanding the recent therapeutic advances [4]. High-risk patients often experience a relapse that occurs early after chemotherapy. Such a relapse of tumors is associated with more aggressive growth, resistance to chemotherapy, and wide metastasis [5]. Neuroblastoma harbors a variety of genetic changes, including a high frequency of MYCN amplification, loss of heterozygosity at 1p36 and 11q, and gain of genetic material from 17q [6]. The MYCN gene codes for the N-myc proto-oncogene protein, a transcription factor. MYCN amplification has been found in 25% of neuroblastoma cases and tags high-risk neuroblastoma, metastatic progression, and poor prognosis [7]. ALK mutations are among the leading cause of hereditary neuroblastoma and account for more than 14% of the somatically acquired alterations [8]. Anaplastic lymphoma kinase (ALK) amplification is found in 2–3% of neuroblastoma and occurs frequently together with MYCN amplification [9]. ALK is a tyrosine kinase receptor belonging to the insulin receptor superfamily. ALK has been considered for a long time as an orphan receptor. Independent studies have nominated ALKAL1 and ALKAL2 as bona fide endogenous ligands of ALK [10,11]. ALK activates multiple signaling pathways and eventually triggers MYCN expression via ALK, PI3K/AKT, MEKK3, MEK5, and ERK5 [12,13]. Conversely, ALK is a direct transcriptional target of MYCN [14]. Such ALK-MYCN positive feedback loops converge on sustained tumor growth [15,16]. Gain-of-function mutations within the ALK kinase domain—F1174, F1245, and R1275—represent around 85% of ALK mutations in neuroblastoma. ALK kinase activity is currently one of the main targets for pharmacological strategies [17]. Unfortunately, resistance to ALK inhibition arises during the therapy. The targeted degradation of oncoproteins can overcome the adaptation to therapeutic agents. Proteolytic approaches, such as Proteolysis-targeting chimera (PROTAC), have now entered clinical testing [18]). PROTAC are small molecules that are able to bind the target and an E3 ubiquitin ligase. Eventually, the PROTAC stimulates the degradation of the target proteins [19]. We leveraged a potential oncosuppressor protein, the IgLON member Negr1, to identify peptides able to induce ALK degradation. IgLONs are membrane-bound proteins that are highly expressed in neurons and involved in cell-to-cell adhesion [20]. IgLONs include three Ig domains and are tethered to the plasma membrane via a GPI link. The expression of the IgLON family results altered in sporadic epithelial ovarian tumors, lung tumors, glioma, and in metastatic neuroblastomas [21,22,23,24,25]. Negr1 modulates neurite outgrowth and synapse establishment [26,27,28,29]. Negr1 is downregulated in various human cancers, including colon, liver, lung, ovary, stomach, and pancreas tumors [30]. Here we describe that Negr1-derived peptides induce ALK downregulation and reduce neuroblastoma progression in vitro and in vivo.

## 2. Materials and Methods

### 2.1. Cell Cultures and Transfection

N2a (Neuro2a, ATCC CCL-131), HEK293 (ATCC CRL-1573), U87 MG (ATCC^®^ HTB14), SKMEL5 (ATCC^®^ HTB70), MCF7 ATCC^®^ HTB-22, A549 ATCC^®^ CCL-185, and SHSY5Y (ATCC^®^ CRL 2266™) cells were grown in DMEM high glucose (Gibco, Thermo Fisher Scientific, Waltham, MA, USA) with 10% FBS, 1% penicillin/streptomycin, and 1% glutamine in a humidified atmosphere of 5% CO_2_ at 37 °C. In the soft-agar assays, we added 0.6% soft agar (Gibco, Thermo Fisher Scientific, Waltham, MA, USA) in media to 2 × 10^3^ cell/cm^2^ N2a cells to obtain a final concentration of 0.4% agar. We cultured the cells onto a 0.6% soft agar layer. N2a and HEK293 cells were transfected with the different constructs for 48 h using Lipofectamine 2000 (Invitrogen, Thermo Fisher Scientific Waltham, MA, USA). Stable N2a clones expressing murine Negr1 were isolated upon Neomycin selection (1 μg/mL).

### 2.2. Constructs and Peptides

Murine Negr1 cDNA (Addgene clone C3342IRCKp5014P057-rzpdm13-21) was cloned into a strep-FLAG pcDNA3.1 vector. mNegr1-peptides (Table 1) and GFP expressing constructs were obtained by PCR and subcloned into a strep-FLAG pcDNA3.1 vector via the Gateway system (Invitrogen, Thermo Fisher Scientific Waltham, MA, USA) using the primers listed below (Table 2). Synthetic PepA-1 peptides were purchased at Genescript (Rijswijk, The Netherlands).

### 2.3. Purification on STREP-Resin

HEK293 cells transfected with the relative constructs were lysed in RIPA buffer (150 mM NaCl, 50 mM HEPES, 0.5% NP40, 1% Sodium-deoxycholate) for one hour at 4 °C and then processed for streptavidin immunoprecipitation. Proteins were eluted from Strep-Tactin Sepharose resin (IBA Lifescience, Göttingen, Germany) in elution buffer (2.5 mM Desthiobiotin, 100 mM Tris-HCL, 150 mM NaCl, 1 mM EDTA) in mild agitation for one hour at 4 °C. The protein concentration was measured via standard Bradford assay (Bio-Rad, Hercules, CA, USA), and protein purity was assessed by SDS-PAGE followed by silver staining.

### 2.4. MTT Assay

We performed the 3-(4,5-dimethylthiazol-2-yl)-2,5-diphenyltetrazolium bromide (MTT) assay to appreciate culture vitality. We cultured N2a cells in a 96-well plate at a concentration of 2 × 10^3^ cells/cm^2^. The treatment condition is indicated in the Results Section 3. At the end of the treatment, we added the MTT solution to the cell medium at a final concentration of 0.25 mg/mL. The incubation lasted 30 min at 37 °C. Next, we removed the medium and collected the formazan precipitates in DMSO (200 μL). We measured the absorbance at 570 nm using a spectrophotometer (Varioskan LUX, Thermofisher, Waltham, MA, USA) as a proxy for cell viability. We expressed relative cell viability fold over the control condition.

### 2.5. Western Blotting

We assessed protein level and relative phosphorylation by Western blotting as in [31]. Briefly, upon a wash in PBS, we solubilized cells and tumor specimens in lysis buffer (150 mM NaCl, 50 mM HEPES, 0.5% NP40, 1% sodium-deoxycholate). After 1 h under mild agitation, we clarified the lysate by centrifugation for 20 min at 16,000× *g*. We performed all experimental procedures at 4 °C. We evaluated protein concentrations via Bradford assay (Bio-Rad, Hercules, CA, USA). For Western blotting experiments, we diluted an equal amount of proteins with 0.25% 5× Laemmli buffer. We separated the samples on 10% SDS-PAGE gels and transferred them onto a nitrocellulose membrane (Sigma-Aldrich) at 80 V for 120 min at 4 °C. Primary antibodies were:

Beta-actin (Santa Cruz Biotechnology, Dallas, TX, USA, sc-47778), ALK (Santa Cruz Biotechnology, Dallas, TX, USA, sc-398791), N-myc (Santa Cruz Biotechnology, Dallas, TX, USA, sc-56729), p44/42 MAPK (Erk1/2) (137F5) Rabbit mAb (Cell Signaling Technology, Danvers, MA, USA), Akt (pan) (11E7) Rabbit mAb (Cell Signaling Technology, Danvers, MA, USA), Phospho-Akt (Ser473) (D9E) XP^®^ Rabbit mAb (Cell Signaling Technology, Danvers, MA, USA), S6 Ribosomal Protein (5G10) Rabbit mAb (Cell Signaling Technology, Danvers, MA, USA), and Negr1 (AF5394) Goat polyclonal (R&D system, Minneapolis, MN, USA). Antibodies were applied overnight in blocking buffer (20 mM Tris, pH 7.4, 150 mM NaCl, 0.1% Tween 20, and 5% nonfat dry milk). We detected proteins using the ECL prime detection system (GE Healthcare, Chicago, IL, USA) with the imaging ChemiDoc Touch system (Bio Rad, Hercules, CA, USA). We quantified the optical density of the specific bands with ImageLab 3.0 software (Bio Rad, Hercules, CA, USA).

### 2.6. RT-qPCR

Total RNA was isolated from cell cultures using a Single Cell RNA Purification Kit (Norgen Thorold, ON, Canada) according to manufacturer’s instruction. After extraction, the RNA concentration was quantified with a NanoDrop 2000C spectrophotometer (Thermo Fisher Scientific, Waltham, MA, USA). After DNAse treatment (Thermo Fisher Scientific, Waltham, MA, USA, according to manufacturer’s protocol), complementary DNA (cDNA) was generated using qRT SuperMix (Bimake). The cDNAs were used for quantitative PCR (qPCR) using iTaq Universal SYBR^®^ Green Supermix and CFX96 RealTime System (BioRad, Hercules, CA, USA) for 40 cycles. We used two independent couples of primers to assess mRNA expression levels (Table 3). All samples were measured as technical duplicates. We calculated mRNA expression levels as the average between the two couple of primers, normalized for the mathematical mean of the relative abundance of actinB and GAPDH (housekeeping genes). Data shown were produced using Bio-Rad CFX Manager 3.1 software and analyzed according to the ddCt method [32].

### 2.7. Orthotopic Implantation of N2a Cells

One million N2a cells were suspended in 150 μL of DMEM high glucose and delivered subcutaneously above the left rear paw in 8-week old nu/nu female mice (Charles River, Wilmington, MA, USA) by injection with a micro-syringe (Hamilton). Negr1-derived peptides were diluted in sterile PBS at the desired concentration and either injected into the center of the tumor mass or intraperitoneally. We performed tumor in situ injection when the tumor was palpable, i.e., it reached 100 mm^3^ volume. We calculated tumor volume using the ellipsoid formula (length × width × height × 0.52). Upon sacrifice, the tumor mass was surgically removed, washed in sterile PBS, and stored at −80 °C. All procedures involving animals were approved by Institutional and National Agencies (authorization 559/2016-PR).

### 2.8. Statistical Analysis and Guidelines

All data are plotted as box, with minimum, maximum, and median indicated. The normality of data distributions was determined using the D’Agostino and Pearson omnibus normality test, followed by an unpaired Student’s *t*-test, ANOVA followed by Tuckey’s post-hoc test, or two-way ANOVA followed by Bonferroni or Student’s t post-hoc test as appropriate. The indication of the number of experiment (n) and level of significance (*p*) are indicated throughout the text. All methods were performed in accordance with the relevant guidelines and national regulations.

## 3. Results

### 3.1. Negr1 Overexpression Halts Cancer Growth In Vitro and In Vivo

**Figure 1:**
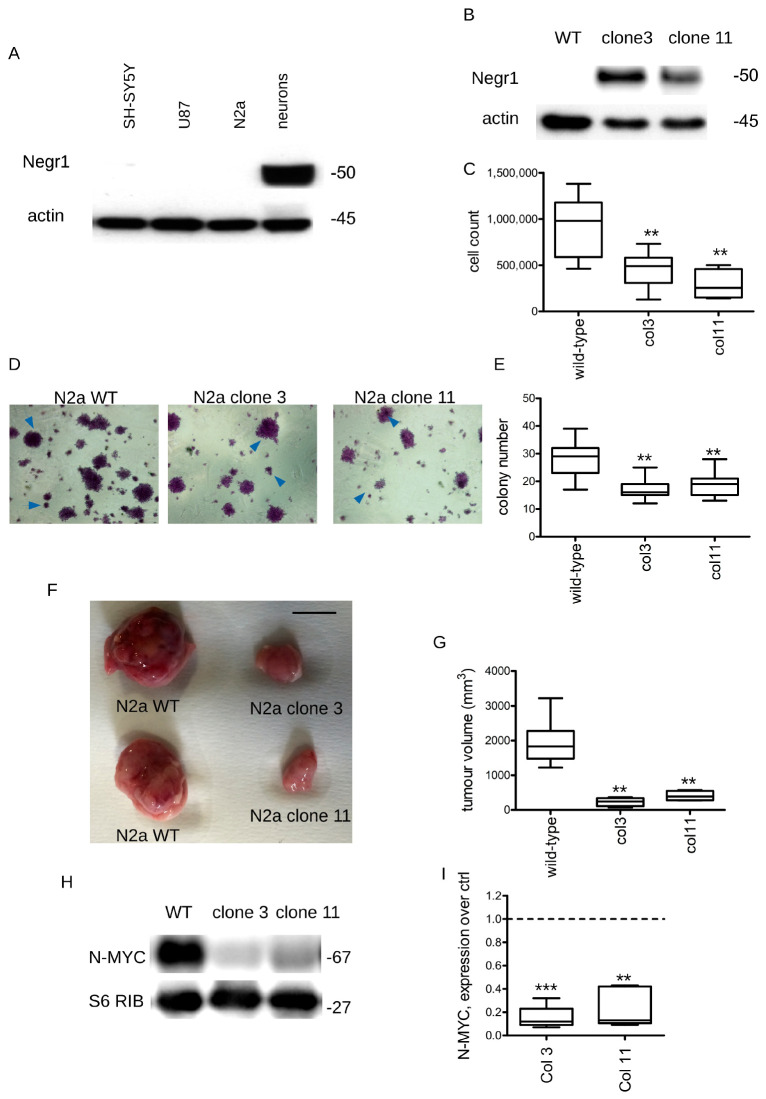
Negr1 overexpression halts neuroblastoma growth in vitro and in vivo. (**A**) Western blot analysis of human neuroblastoma (SH5Y), glioblastoma (U87), murine neuroblastoma (N2a), and murine primary cortical neurons. Samples were stained with anti-Negr1 and anti-actin antibodies. (**B**) We generated stable N2a clones overexpressing Negr1. We validated Negr1 expression by Western blotting. (**C**) We analyzed the proliferation rate of wild-type N2a cells and of two different clones stably expressing Negr1 (colonies 3 and 11). N2a clones were seeded at a fixed amount (2 × 10^3^ cells/cm^2^) at day 0. At DIV5, we counted cell number; n = 12, ** *p* < 0.01 vs. wild-type. (**D**) We seeded 2 × 10^3^ cells/cm^2^ N2a wild-type or stably expressing Negr1 (col3 and 11) cells in soft-agar and cultured them for 14 days. Next, we stained cells with crystal violet. (**E**) The graph reports the number of colonies (cluster encompassing more than 50 cells); n = 6, ** *p* < 0.01 vs. wild-type. (**F**) We subcutaneously injected 1 million N2a cells (wild-type, clone 3, and clone 11) in CD1 immunodeficient nude mice. We monitored tumor growth on days 3, 5, 7, 9, 13, and 15 after injection. The image shows representative tumor masses, scale bar = 1 cm. (**G**) The graph reports tumor volume; n = 6, ** *p* < 0.01 vs. wild-type. (**H**) We analyzed tumors by biochemical means. (**I**) The graph reports N-MYC levels, normalized over S6RP amount, and expressed as fold over wild-type derived tumors (ctrl), ** *p* < 0.01, *** *p* < 0.001 vs. wild-type.

The IgLON family member Negr1 has been identified as a commonly downregulated gene in many human cancer tissues [30,33]. Accordingly, we observed that the Negr1 protein is downregulated in cancer-derived cell lines. In particular, we processed via Western blotting protein samples obtained by human neuroblastoma (SH5Y), glioblastoma (U87), murine neuroblastoma (N2a) lines, and murine primary cortical neurons. The Negr1 protein level was below detection in all samples derived from cancer lines (Figure 1A). Next, we assessed whether Negr1 protein levels correlate with cellular proliferation. To this aim, we generated and characterized two N2a clones expressing the Strep-FLAG Negr1 fusion protein (Figure 1B). At day in vitro 0 (DIV0), we seeded 2 × 10^3^ cells/cm^2^ cells obtained from wild-type cells, clone 3, and clone 11. At DIV5, we evaluated cellular proliferation. We observed that ectopic Negr1 expression significantly reduced N2a proliferation (Figure 1C). Cancer cells gain the capability to grow independently of a solid surface. Thus, we monitored the growth of N2a clones on a soft agar matrix [34]. By scoring the number of colonies at DIV 14 (cluster encompassing more than 50 cells), we noticed that Negr1-expressing N2a cellular clones generated few colonies on soft agar (Figure 1D,E). Xenografts in immunodeficient mice represent an established tool to investigate cancer progression in vivo [35]. We subcutaneously injected (s.c.) 1 million N2a cells (naive, clone 3, and clone 11) in CD1 nude mice. We monitored tumor growth ex vivo by measuring tumor size with a Vernier caliper. We noticed that the tumors derived from Negr1-expressing cells were significantly smaller than the tumors derived from wild-type N2a cells (Figure 1F,G). The altered activity of the ALK receptor and increased expression of the downstream MYC transcription factor are the pivotal molecular hallmarks of neuroblastoma. Thus, we monitored the N-MYC protein level in specimens gathered from tumors generated by wild-type and Negr1-expressing cells (Figure 1H). By Western blotting, we observed a significant reduction in the N-MYC protein level in Negr1-expressing tumors (Figure 1I).

### 3.2. Negr1 Impacts ALK Protein Levels

**Figure 2:**
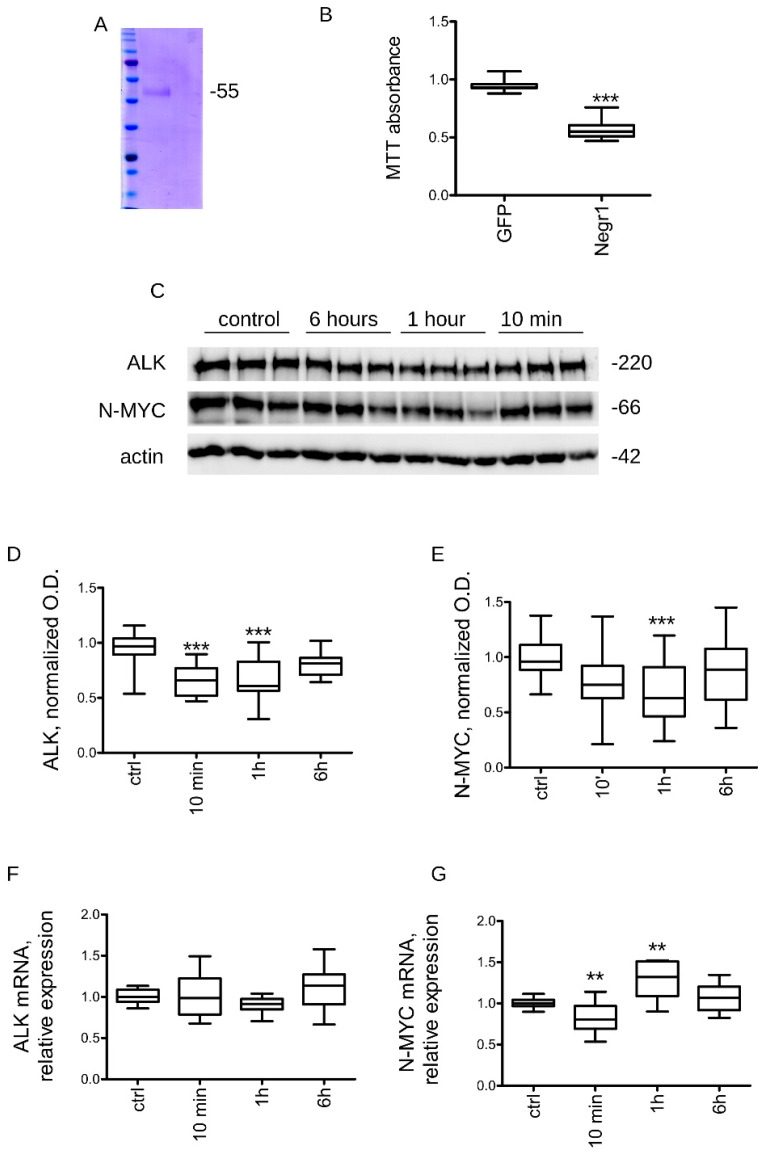
Negr1 halts N2a growth and induces ALK degradation. (**A**) We purified Negr1 (rNegr1) upon expression in HEK293 cells. We monitored protein yield and purity by Coomassie staining upon SDS-PAGE. (**B**) We analyzed the proliferation rate of wild-type N2a cells treated with GFP or rNegr1 (100 ng/mL, daily). N2a clones were seeded at a fixed amount (2 × 10^3^ cells/cm^2^) at day 0 and cultured for 5 days. We measured culture viability by MTT assay. The graph reports cell viability expressed as fold over not treated culture; n = 10, ** *p* < 0.01 vs. GFP. (**C**) We treated 5 × 10^3^ cells/cm^2^ N2a cells with 100 ng/mL GFP or rNegr1 for 10 min or 1 or 6 h. Next, we processed the culture for Western blotting or RT-qPCR purposes. (**D**,**E**) The graphs report ALK (**D**) and N-MYC (**E**) protein levels, normalized over actin; n = 6, *** *p* < 0.001 vs. GFP (ctrl). (**F**,**G**) The graphs report ALK (**F**) and N-MYC (**G**) mRNA levels, normalized over housekeeping genes; n = 6, ** *p* < 0.01 vs. ctrl.

Negr1 exists as a GPI-anchored membrane-bound protein and as a soluble protein released upon metalloprotease cleavage [36]. Soluble Negr1 promotes neurite outgrowth in primary neurons [28]. Thus, we assessed whether ectopic treatment with the Negr1 protein might modulate cancer cell growth. To this aim, we expressed and purified from HEK293 cells Strep-FLAG Negr1 (rNegr1) (Figure 2A). Next, we treated 2 × 10^3^ cells/cm^2^ naive N2a cells daily with 100 ng/mL rNegr1 or GFP, a biologically inert protein, used as control. At DIV5, we evaluated cellular growth by MTT assay. We observed that the treatment with Negr1 significantly reduced N2a growth (Figure 2B). Subsequently, we monitored whether treatment with GFP (control) or Negr1 might impact ALK or N-MYC expression. To this aim, we treated 5 × 10^3^ cells/cm^2^ N2a cells with 100 ng/mL GFP or rNegr1 for different times, ranging from 10 min to 6 h (Figure 2C). At the end of the treatment, we processed the culture to assess ALK and N-MYC expression at the protein and mRNA levels. Treatments with Negr1 for up to one hour induced a reduction of ALK and MYCN proteins (Figure 2D,E). Interestingly, we did not notice an overt impact of rNegr1 on ALK mRNA levels (Figure 2F). Instead, rNegr1 had a light but significant effect on N-MYC mRNA levels (Figure 2G).

### 3.3. Negr1-Derived Peptides Halt Cancer Growth In Vitro

**Figure 3:**
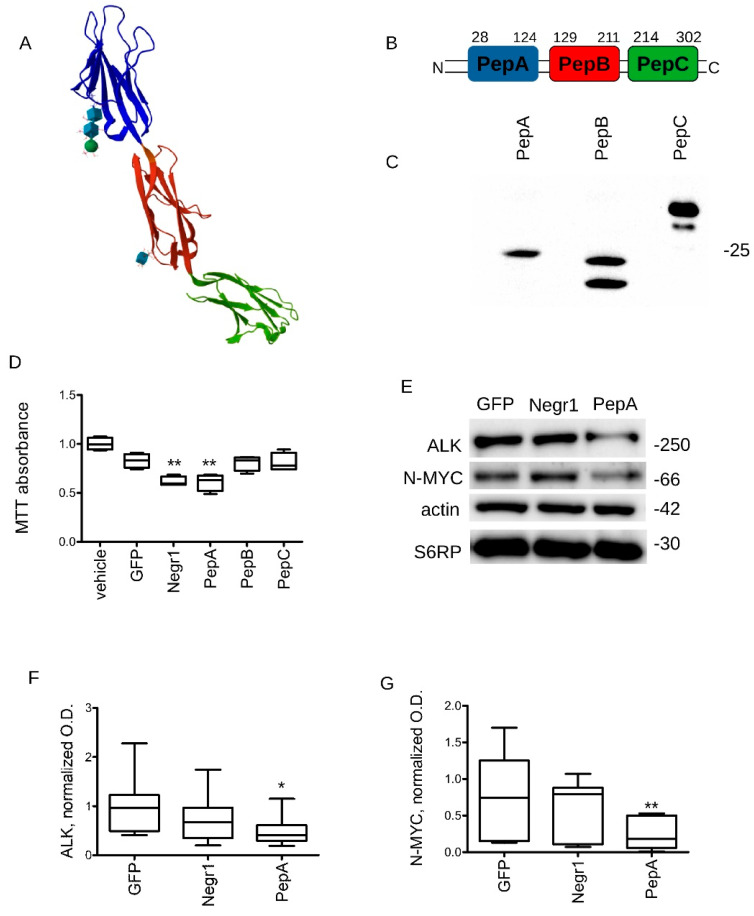
PepA halts N2a growth and induces ALK degradation. (**A**) The model shows a Negr1 monomer (adapted from PDB: 6U6T). The three Ig domains are represented in blue, red, and green. (**B**) The cartoon depicts the boundaries of three peptides that we generated, encompassing the three Negr1 Ig domains. (**C**) We purified the three different peptides upon expression in HEK293 cells. We monitored peptide expression by Western blotting using an anti-FLAG antibody. (**D**) We analyzed the proliferation rate of wild-type N2a cells treated with GFP, rNegr1, or Negr1-derived PepA, PepB, and PepC (100 ng/mL, daily). N2a cells were seeded at a fixed amount (2 × 10^3^ cells/cm^2^) at day 0 and cultured for 5 days. The graph reports cell number at day 5; n = 12, ** *p* < 0.01 vs. GFP. (**E**) We treated 2 × 10^3^ cells/cm^2^ N2a cells with 100 ng/mL GFP, rNegr1, or PepA daily for 5 days. At DIV5, we processed the culture for Western blotting and we evaluated the protein expression level for ALK, N-MYCN, actin, and S6 ribosomal protein. (**F**,**G**) The graphs report ALK (**C**) and MYC (**D**) levels, normalized over actin and S6RP amount and expressed as fold over GFP treatment; n = 6, * *p* < 0.05 vs. GFP.

The structure of Negr1 has been solved by X-ray diffraction (Figure 3A, adapted from PDB: 6U6T) [37]. To identify the minimum sequence capable of executing Negr1-antineoplastic action, we cloned, expressed, and purified from HEK293 cells each Ig domain as a strep-FLAG tagged recombinant protein, hereinafter called PepA, PepB, PepC (Figure 3B,C). We treated 2 × 10^3^ cells/cm^2^ naive N2a cells daily with the three domains. At DIV5, we evaluated cellular growth by MTT assay. We observed that the treatment with Negr1 and PepA significantly reduced N2a proliferation (Figure 3D). Subsequently, we monitored whether treatment with rNegr1 or PepA might impact ALK and N-MYC expression. To this aim, we treated 2 × 10^3^ cells/cm^2^ N2a cells with 100 ng/mL GFP, rNegr1, or PepA daily for five days. At DIV5, we processed the culture for Western blotting and we evaluated ALK and N-MYC protein expression levels. PepA treatment induced a robust reduction of ALK and N-MYCN protein levels (Figure 3E–G).

**Figure 4:**
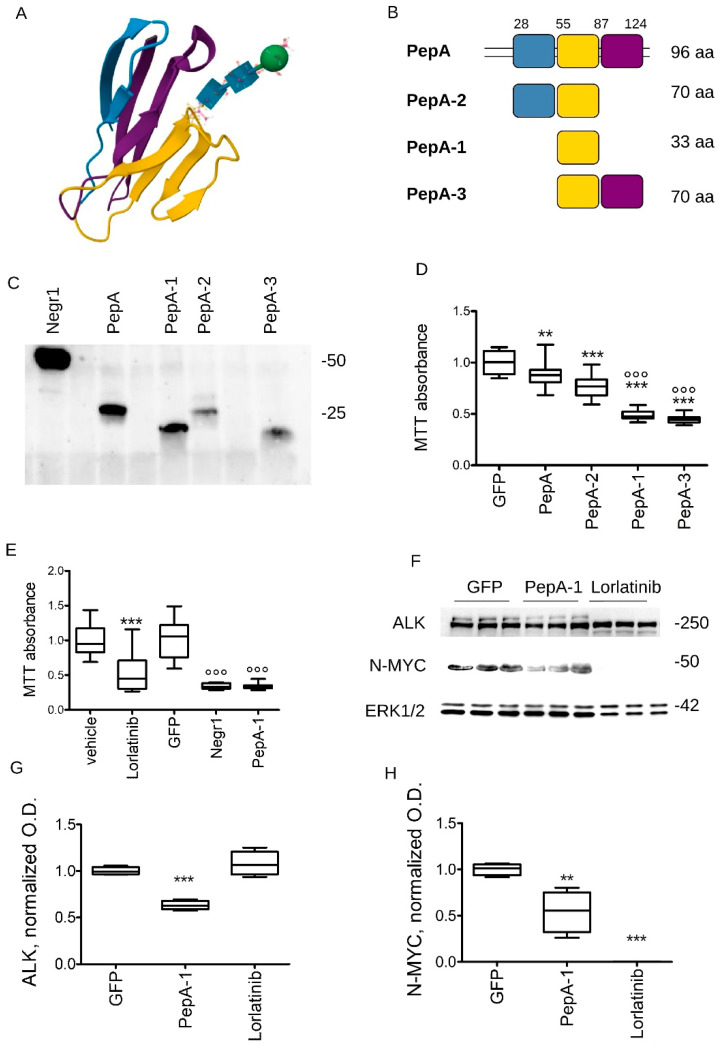
Soluble Negr1-derived peptides halt neuroblastoma growth in vitro and in vivo. (**A**) The model shows the structure of the first Ig domain, included in PepA (adapted from PDB: 6U6T). We isolated three different regions in PepA, represented in blue, purple, and yellow. (**B**) The cartoon depicts the boundaries of the PepA-derived peptides. (**C**) We purified the three different peptides upon expression in HEK293 cells. We monitored peptides expression by Western blotting using an anti-FLAG antibody. (**D**) We analyzed the proliferation rate of wild-type N2a cells treated with recombinant GFP, PepA, PepA-1, PepA-2, and PepA-3 (100 ng/mL, daily). N2a cells were seeded at a fixed amount (2 × 10^3^ cells/cm^2^ cells) at day 0 and cultured for 5 days, and eventually measured by MTT assay. The graph reports cell vitality at day 5 normalized to GFP value; n = 10, ** *p* < 0.01 vs. GFP. (**E**) We analyzed the proliferation rate of wild-type N2a cells treated with vehicle, Lorlatinib, GFP, rNegr1, and PepA-1 (3 μM, daily). N2a cells were seeded at fixed amount (2 × 10^3^ cell/cm^2^) at Day 0 and cultured for 5 days, and eventually measured by MTT assay. The graph reports cell vitality at day 5 normalized to values measured upon vehicle (for Lorlatinib) or GFP (for rNegr1, and PepA-1) treatment; n = 10, *** *p* < 0.01 vs. vehicle, °°° *p* < 0.01 vs. GFP. (**F**) We treated 2 × 10^3^ cells/cm^2^ N2a cells with GFP, Lorlatinib, or PepA-1 (3 μM, daily). At DIV5, we processed the culture for Western blotting and we evaluated the protein expression level for ALK, N-MYC, and actin. (**F**,**G**) The graphs report ALK (**G**) and N-MYC (**H**) levels, normalized over ERK1/2 and expressed as fold over cell treated with GFP; n = 6, ** *p* < 0.01, *** *p* < 0.001 vs. GFP.

PepA encompasses multiple beta-strands (Figure 4A, adapted from PDB: 6U6T). To further narrow down the minimal biological active sequence within PepA, we cloned and characterized three peptides covering the PepA sequence and respecting its structural features (Figure 4B). We expressed and purified from HEK293 cells the three peptides as strep-FLAG tagged peptides, hereinafter called PepA-1, PepA-2, and PepA-3 (Figure 4C). To assess the in vitro activity of the three peptides, we seeded 2 × 10^3^ cells/cm^2^ naive N2a cells and treated them daily with 100 ng/mL GFP, PepA, PepA-1, PepA-2, and PepA-3. At DIV5, we evaluated cellular growth by MTT assay. We observed that the minimal peptide PepA-1 significantly reduced N2a growth (Figure 4D). To further examine the efficacy of PepA1, we investigated N2A cell proliferation upon chronic treatment with vehicle, Lorlatininb (an FDA-approved ALK inhibitor), GFP, rNegr1, and PepA-1 (daily, all molecules at 3 μM). At DIV5, we evaluated cellular vitality by MTT assay. rNegr1, PepA-1, and Lorlatinib impaired N2A growth to a similar extent (Figure 4E). Finally, we treated 2 × 10^3^ cells/cm^2^ N2a cells with GFP, Lorlatinib, or PepA-1 (all at 3 μM) daily for five days and then we evaluated ALK and N-MYC protein expression by Western blotting. The chronic treatment with PepA-1 triggered a downregulation of both ALK and N-MYC, while Lorlatinib strongly reduced only N-MYC levels (Figure 4F–H).

### 3.4. Soluble Negr1-Derived Peptides Halt Cancer Growth In Vivo

**Figure 5:**
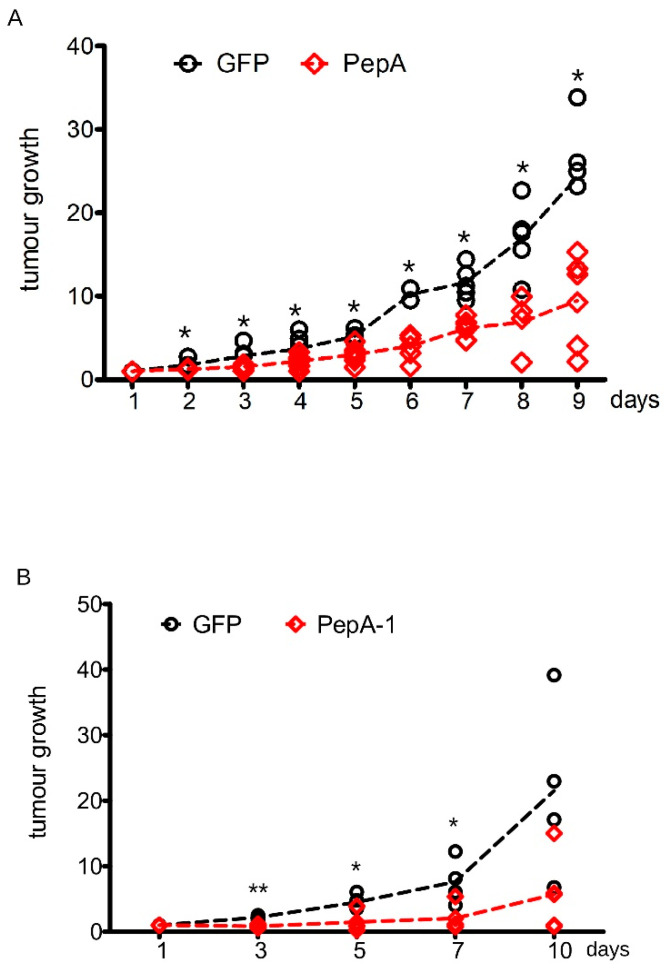
PepA halts neuroblastoma growth in vivo. (**A**) We subcutaneously injected 1 million N2a cells in CD1 immunodeficient nude mice. When the tumor reached 100 mm^3^ volume, we injected 2 ug of GFP or PepA in situ. We repeated the treatment every second day. We monitored tumor growth from day 1 to 9 after injection. The graph reports tumor volume growth expressed as fold over day 1; n = 9, * *p* < 0.05 vs. GFP. (**B**) We subcutaneously injected 1 million N2a cells in CD1 immunodeficient nude mice. When tumors reached 100 mm^3^ volume, we injected 2 ug of GFP or PepA-1 in situ. We repeated the treatment every second day. We monitored tumor growth on day 3, 5, 7, and 10 after injection. The graph reports tumor volume growth expressed as fold over day 1; n = 5, * *p* < 0.05, ** *p* < 0.01 vs. GFP.

**Figure 6:**
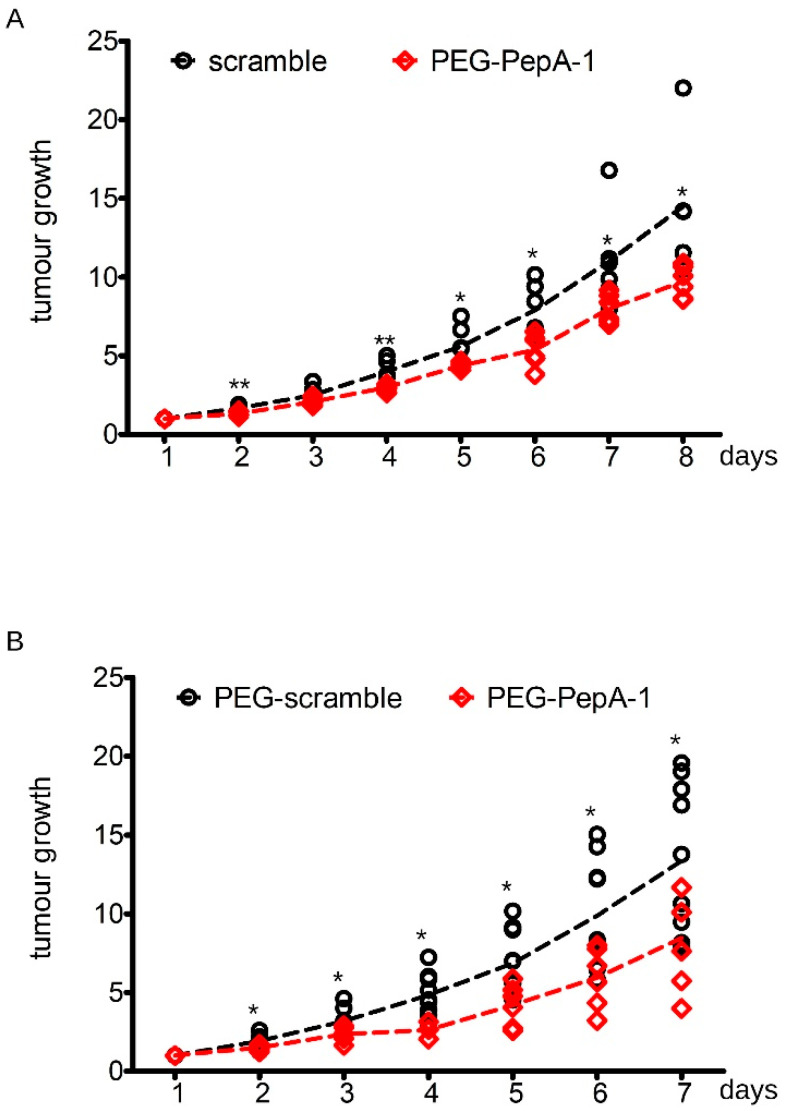
Synthetic PEG-PepA1 halts neuroblastoma growth in vitro and in vivo. (**A**) We subcutaneously injected 1 million N2a cells into CD1 immunodeficient nude mice. When tumors reached 100 mm^3^ volume, we injected 10 ug of PEG-scramble peptide or PEG-PepA-1 in situ. We repeated the treatment every second day. We monitored tumor growth daily after injection. The graph reports tumor volume growth expressed as fold over day 1; n = 5, * *p* < 0.05, ** *p* < 0.01 vs. PEG-scramble. (**B**) We subcutaneously injected 1 million N2a cells into CD1 immunodeficient nude mice. When tumors reached 100 mm^3^ volume, we injected 150 ug of PEG-scramble peptide or PEG-PepA-1 intraperitoneally. We repeated the treatment every second day. We monitored tumor growth daily after injection. The graph reports tumor volume growth expressed as fold over day 1; n = 6, * *p* < 0.05 vs. PEG-scramble.

To further assess the anti-neoplastic activity of Negr1-derived peptides, we injected s.c 1 million N2a cells into 6-week-old CD1 nude mice. When the tumor reached 100 mm^3^ volume, we injected 2 ug of GFP, PepA, or PepA-1 in situ. We repeated the treatment every second day and monitored tumor growth daily. We observed a robust growth reduction in tumors upon PepA and PepA-1 treatment (Figure 5A,B). Database prediction suggests that PepA1 is glycosylated on the asparagine residue (N) in position + 10. We designed a synthetic peptide encompassing the PepA-1 sequence coupled at the N-terminus to a mini-PEG1 moiety (MW: 152 Da) to improve the pharmacokinetic properties of the peptide [38,39]. The resulting peptide, hereinafter PEG-PepA-1, is soluble in water at a concentration of 5 mg/mL. To appreciate the anti-neoplastic effect of PEG-PepA-1, we injected s.c. 1 million N2a cells into 6-week-old CD1 nude mice. When the tumor reached 100 mm^3^ volume, we injected 10 ug of PEG-PepA-1 or PEG-scramble peptide in situ. We repeated the treatment every second day and monitored tumor growth daily. We observed a robust growth reduction in tumor growth upon PEG-PepA treatment (Figure 6A). Finally, we tested the efficacy of PEG-PepA-1 upon systemic administration. To this aim, we injected s.c 1 million N2a cells into 6-week-old CD1 nude mice. When the tumor reached 100 mm^3^ volume, we injected 150 ug of PEG-PepA-1 or PEG-scramble peptide intraperitoneally. We repeated the treatment every second day and monitored tumor growth daily. We observed a robust growth reduction in tumor growth upon PEG-PepA-1 treatment (Figure 6B).

## 4. Discussion

Our work proposes Negr1-derived peptide as a new therapeutic opportunity targeting the receptor tyrosine kinase (RTK) ALK. Indeed, both physical and functional interactions link IgLON to RTKs. In particular, OPCML binds to the extracellular domain of HER2 and regulates its clathrin-independent endocytosis [22]. Similarly, we showed that Negr1 binds FGFR2 and regulates its subcellular distribution [26]. It is established that the IgLON family member OPCML acts as a tumor suppressor by eliciting the downregulation or the inhibition of RTKs, such as EphA2, FGFR1, FGFR3, HER2, HER4, and AXL [40]. We reported that the acute treatment with soluble Negr1 induced a rapid reduction of ALK protein levels without affecting mRNA expression, suggesting that Negr1 induced ALK protein degradation. Unexpectedly, the chronic treatment (5 days) with soluble Negr1, while sufficient to halt cellular growth, did not overtly affect ALK or N-MYC protein levels.

This outcome may reflect that Negr1 influences not only ALK levels but also its downstream signaling, involved in cell proliferation. Instead, the chronic treatment with the Negr1-derived PepA was able to slow cellular proliferation and decrease ALK and N-MYC proteins. These observations may suggest the occurrence of compensatory effects upon the treatment with full-length Negr1 only. Indeed, Negr1 homodimerization involves a surface generated by β-strands falling in the first Ig domain, therefore included in PepA and partially in PepA-1 [37]. Recent data showed that Negr1 interacts with the IL-6 receptor mainly via the third Ig domain (included in PepC) [41]. As such, PepA and the derived peptide PepA-1 may self-interact but cannot establish the full Negr1-interactome. Clearly, the supra-molecular complex orchestrated by Negr1 could be relevant for triggering the intracellular events that, eventually, contribute to regulating ALK protein levels and, consequently, MYC-N expression. Another intriguing possibility is that Negr1 binds ALK via a sequence included in PepA-1. Other regions of Negr1 may instead stabilize the receptor on the membrane and spare it from degradation, as we described for FGFR2 [26]. PepA and PepA-1 alone may instead trigger ALK degradation via a ligand-dependent mechanism. Given that ALK activation is pivotal to boost neuroblastoma growth, its degradation may slow tumor progression.

ALK is an ideal target for a selective therapeutic approach. Its expression is limited in normal tissue but high in neuroblastoma [42]. Indeed, ALK can be pharmacologically blocked via ATP-competitive inhibitors of the tyrosine kinase domain. Crizotinib was the first ALK inhibitor approved for non-small cell lung cancer with ALK mutations [43]. A second-generation inhibitor, alectinib, outperformed crizotinib in terms of therapeutic response and became the first-line treatment for non-small cell lung cancer [44]. However, evidence from ALK fusion-positive lung cancer has shown that resistance to ALK inhibition occurs during the therapy, causing a relapse within several years. Such an escape mechanism underlies both the secondary mutation and amplification of ALK and the activation of alternative signaling cascades [45]. Lorlatinib (PF-6463922) inhibits ALK and ROS1, and it has been designed to overcome ALK mutants resistant to other inhibitors [46,47]. Strikingly, preclinical studies demonstrated the efficacy of lorlatininb in reducing neuroblastoma in vitro and in vivo [47,48]. However, point mutations, such as G1202R + L1196M, guarantee resistance even to Lorlatinib treatment [49,50]. A similar mechanism may occur in neuroblastoma patients upon Lorlatinib treatment. Currently, no effective therapies exist against Lorlatinib-resistant disease [51]. Furthermore, the CROWN study highlighted several adverse effects upon Lorlatinib treatment, such as hypercholesterolemia, hypertriglyceridemia, peripheral neuropathy, and cognitive impairments [52]. Clearly, alternative therapeutic strategies are required. The selective degradation of oncoproteins is an attractive approach expanding the druggable proteome and overcoming the molecular adaptation to current therapeutic agents. Targeted proteolysis bears promising potential in therapeutic applications [53]. Current proteolytic approaches rely on the capability to re-route the target to the cellular degradation machinery, encompassing lysosomes and the ubiquitin–proteasome system [54,55].

Proteolysis-targeting chimera (PROTAC) protein degraders have now entered clinical testing [18]. PROTAC are heterobifunctional small molecules encompassing two functional structures: one binds the protein of interest while the other recruits an E3 ubiquitin ligase. As such, PROTAC induces the ubiquitylation and, eventually, the degradation of the target proteins [19]. However, membrane proteins are currently not considered ideal targets for PROTAC therapy [56]. The Negr1-derived peptide described here demonstrated the capability to degrade ALK and slow tumor progression in vitro and in vivo.

## 5. Conclusions

Peptides represent a promising class of pharmaceutical compounds with several advantages over proteins or antibodies: they are small in size, easy to synthesize, and can penetrate cell membranes. Indeed, the PEGylated synthetic peptides used in our experiments demonstrated the ability to distribute from the injection site to the tumor mass, as demonstrated by the tumor reduction elicited upon systemic administration. Furthermore, it is feasible to explore the biological and chemical diversity to design peptides with high specificity, affinity, and minimal drug–drug interaction. Clearly, the peptide described here requires development to improve its pharmacodynamics and pharmacokinetics properties further, but it may represent a promising therapeutic tool for the treatment of aggressive neuroblastoma resistant to current ALK inhibitors.

## Figures and Tables

**Table 1:** Sequence of the peptides described in the manuscript.

Peptide Name	Sequence
PepA	VDFPWAAVDNMLVRKGDTAVLRCYLEDGASKGAWLNRSSIIFAGGDKWSVDPRVSISTLNKRDYSLQIQNVDVTDDGPYTCSVQTQHTPRTMQVHL
PepB	PKIYDINMTINEGTNVTLTCLATGPEPVISWRHISPSAKPFENGQYLDIYGITRDQAGEYECSAENDVSFPDVKKVRVI
PepC	APTIQEIKSGTVTPGRSGLIRCEGAGVPPPAFEWYKGEKRLFNGQQGIIIQNFSTRSILTVTNVTQEHFGNYTCVAANKLGTTNASLP
PepA1	DGASKGAWLNRSSIIFAGGDKWSVDPRVSISTL
PepA2	VDFPWAAVDNMLVRKGDTAVLRCYLEDGASKGAWLNRSSIIFAGGDKWSVDPRVSISTL
PepA3	DGASKGAWLNRSSIIFAGGDKWSVDPRVSISTLNKRDYSLQIQNVDVTDDGPYTCSVQTQHTPRTMQVHL

**Table 2:** Sequence of the primers used to clone Negr1-derived peptides.

Primer	Sequence (5′-3′)
Negr1 FW	GTGCTCCTGGCGCAGGGC
Negr1 REV	TAAGATGCAGACCAGAAG
PepA FW	GTGGACTTCCCTTGG
PepA REV	GAGATGAACCTGC
PepB FW	CCGAAAATATACG
PepB REV	GATCACTCTCAC
PepC FW	GCGCCTACAATTCAG
PepC REV	GGGCAGGCTCGCGTTG
PepA1 FW	GATGGAGCATCAAAG
PepA1 REV	CAATGTGGAAATG
PepA2 FW	GTGGACTTCCCTTG
PepA2 REV	CAATGTGGAAATG
PepA3 FW	GATGGAGCATCAAAG
PepA3 REV	GAGATGAACCTGC

**Table 3:** Sequence of the primers used in the RT-qPCR analyses.

Primer	Sequence (5′-3′)
ALK1 FW	ACTGACACTCTCGCTTCTGAA
ALK1 REV	ATACGTTTCCTCTCAAAACCCC
ALK2 FW	GCTCCATGGAGTCACCCTC
ALK2 REV	CTCGAGGCCTCCTCGGAG
NMYC1 FW	GGTGGCTGCTCCTGCTCGTG
NMYC1 REV	TCCTCTTCATCTTCCTCCTCGT
NMYC2 FW	GCGGTCACTAGTGTGTCTG
ActinB FW	AATCGTGCGTGACATCAAAG
ActinB REV	AAGGAAGGCTGGAAAAGAGC
Gapdh FW	ACCACAGTCCATGCCATC
Gapdh REV	CAGGAAATGAGCTTGACAAAG

## Data Availability

The full data set is available upon reasonable request.

## Abbreviations

ALK	Anaplastic lymphoma kinase
col	Colony
ctrl	Control
DIV	Day in vitro
IgLON	Immunoglobulin LSAMP, OBCAM, Neurotrimin family
MTT	3-(4,5-dimethylthiazol-2-yl)-2,5-diphenyltetrazolium bromide
rNegr1	Recombinant Strep-FLAG Negr1
RTK	Receptor tyrosine kinase
S.C.	Subcutaneous
WT	Wild-type
